# Supplementary material for: Age and Sex Determine Electrocardiogram Parameters in the Octodon degus
Source: Biology (Basel). 2023 May 19;12(5):747. doi: 10.3390/biology12050747 (PMC10215068; doi:10.3390/biology12050747)
Supplement: Supplementary file 1 [file biology-12-00747-s001.zip › biology-2319758-supplementary.pdf]

Supplementary Material

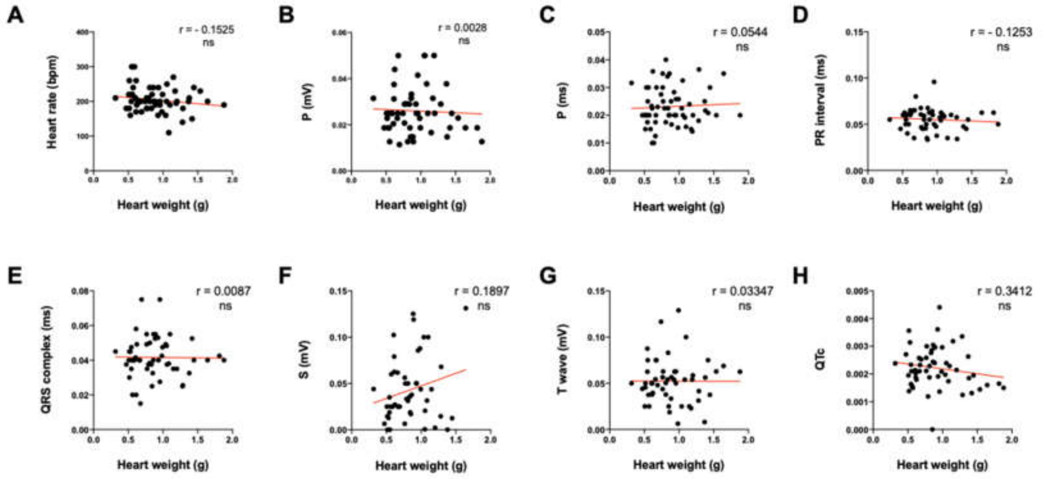

Supplementary Figure S1. Correlations between heart weight and ECG parameters. Correlations between all heart weight (g) and the different ECG parameters. Data are expressed as individual values in the correlations, with red line indicating linear regression. Number of XY pairs = 55. Asterisks indicate the significance of the statistical differences: ** $p<0.01$, **** $p<0.0001$. Abbreviations: bpm = beats per minute; ms = milliseconds; ns = not significant; r = Spearman's coefficient.
